# Factors associated with access to healthcare in Burkina Faso: evidence from a national household survey

**DOI:** 10.1186/s12913-021-06145-5

**Published:** 2021-02-15

**Authors:** Hilaire Zon, Milena Pavlova, Wim Groot

**Affiliations:** 1 National Laboratory of Public Health, Ministry of Health, PO Box 6753, Ouagadougou, Burkina Faso; 2 Department of Health Services Research, CAPHRI, Maastricht University Medical Centre, Faculty of Health, Medicine and Life Sciences, Maastricht University, Maastricht, The Netherlands; 3 Top Institute Evidence-Based Education Research (TIER), Maastricht University, Maastricht, The Netherlands

**Keywords:** Healthcare, Access, Household survey, Burkina Faso, Accès aux Soins, Enquête Ménages, Burkina Faso

## Abstract

**Background:**

Burkina Faso has undertaken major reforms, the cornerstone of which has been the decentralization of the health system to increase access to primary healthcare and to increase the effectiveness, efficiency, financial viability and equity of health services. This study aims to analyze the socio-demographic determinants of households’ access to healthcare in Burkina Faso.

**Methods:**

We used data from a national household survey conducted in 2014 in Burkina Faso. We carried out binary logistic and linear regression analysis using data from a national household survey. The statistical analysis explored the associations between socio-demographic characteristics on the one side, and the use of health services, satisfaction with health services and expenditures on health services, on the other side.

**Results:**

The findings indicate an association between age, education, income and use of services (*p* < 0.0005). The results show that healthcare users’ satisfaction is influenced by age, the association is stronger with the age group under 24 (*p* < 0.0005) than the age group of 25–39 (*p* < 0.005). An association was found between the age group under 15 (*p* < 0.005), the type of health facility used (*p* < 0.0005), the distance traveled to health facilities (*p* < 0.005) and households’ individuals’ health expenditure.

**Conclusion:**

Specific policies are needed to enhance geographical access to healthcare, financial access to and satisfaction with healthcare in moving towards universal health coverage (UHC).

**Supplementary Information:**

The online version contains supplementary material available at 10.1186/s12913-021-06145-5.

## Résumé

**Introduction:**

Le Burkina Faso a entrepris des réformes majeures dans le domaine de la santé dont l’une des pierres angulaires a été la décentralisation du système de santé en vue d’accroître l’accès aux soins de santé primaire et d’améliorer l’efficience, l’efficacité, la viabilité financière et l’équité des services de santé. La présente étude vise à analyser les déterminants de l’accès des ménages aux services de santé au Burkina Faso.

**Méthode:**

Pour notre analyse, nous avons utilisé les données secondaires d’une enquête nationale réalisée en 2014 au Burkina Faso sur le profil de pauvreté et d’inégalités des ménages. Une régression logistique binaire et linéaire a été réalisée pour analyser l’association entre les caractéristiques sociodémographiques et l’utilisation des services de santé d’une part, le niveau de satisfaction des utilisateurs envers les services de santé et les dépenses de santé d’autre part.

**Résultats:**

Les résultats indiquent une association entre l’âge, l’éducation, le revenu et l’utilisation des services de santé (*p* < 0.0005). Les résultats montrent que la satisfaction des utilisateurs des services de santé est. influencée par l’âge, la corrélation est. plus forte avec le groupe d’âge de moins de 24 ans (*p* < 0.0005) que le groupe d’âge de 25–39 (*p* < 0.005). Une corrélation a été mise en évidence entre le groupe d’âge de moins de 15 ans (*p* < 0.005), le type de structure de santé utilisé (*p* < 0.0005), la distance parcourue pour le recours aux soins (*p* < 0.005) et les dépenses de santé des ménages.

**Conclusion:**

Des politiques spécifiques sont nécessaires pour améliorer l’accès géographique et financier des populations aux services de santé, ainsi que le niveau de satisfaction des utilisateurs des services de santé dans la perspective de la couverture sanitaire universelle.

## Background

Providing equal access to essential healthcare and financial risk protection are two core purposes of a health system that targets universal health coverage (UHC). Promoting and protecting health is essential to human welfare as well as to sustained economic and social development [34]. Already in 1978, the Alma Ata declaration emphasized the importance of primary healthcare to achieve the health-for-all goal. The declared guiding principles, which are still largely applicable, concern community participation, which is needed to cater to community needs and to set priorities, values and vision for the health system [33].

Based on the Alma Ata declaration and the related policy momentum, several sub-Saharan African countries, including Burkina Faso, launched health system reforms aimed at operationalizing the declaration’s recommendations to improve access to healthcare for all citizens [31]. In some of these countries, the reforms were part of the wider macroeconomic policy and implementation of structural adjustment programs, which required control of public expenditure as well as changes in public and private sector institutional structures [11].

In line with these changes, since the end of the 1980s, Burkina Faso has undertaken major reforms [6], the cornerstone of which has been the decentralization of the health system. This included the creation of health directorates and health districts in 1993, the establishment of autonomy over financial and human resources management for hospitals, and the creation of a central procurement agency for essential medicines. In 1987, Burkina Faso endorsed the Bamako initiative that aimed to increase access to primary healthcare by raising the effectiveness, efficiency, financial viability and equity of health services [30].

The notable elements of this initiative were the introduction of healthcare users’ fees and the establishment of local health committees to enhance community participation in healthcare financing and management.

Despite the different reforms and initiatives undertaken in Burkina Faso, the population continues to face major challenges in access and use of health services. As suggested in the literature, a variety of factors may influence access and use of healthcare, including socio-demographic factors, policy and institutional considerations [1, 2, 4, 9, 13, 27–29, 32, 35]. This study explores the association of household-level factors with access to and utilization of health services in Burkina Faso.

In particular, the study aims to analyze the socio-demographic determinants of households’ access to healthcare in Burkina Faso. We apply quantitative analytical methods to national household survey data to better understand health service utilization in Burkina Faso. The study contributes to a further exploration and explanation of the inequitable access and use of healthcare in Burkina Faso. The study could be useful for policy in Burkina Faso when assessing the reform progress as well as for other countries in the region that struggle with problems in healthcare access.

## Methods

### Study design and dataset

The study was quantitative. We used data from a national household survey conducted in 2014 in Burkina Faso by the National Institute of Statistics and Demography. This survey was designed to be representative of the country and involved a large sample of households selected across all 45 provinces of the country, covering all 13 regions.

### Sampling and sample size

The survey employed a two-stage stratified sampling approach. At the first stage, 900 sampling units were drawn across the provinces with a probability proportional to the size of the population in the provinces. At the second stage, a sample of 12 households was selected at random (with equal probability) from each sampling unit selected at the first stage. For this purpose, the list of households registered during the last census in the 900 sampling units, was used. The sampling procedure resulted in 10,800 households, including 72,401 individuals (household members) who were invited to participate (INSD, [18]).

### Questionnaire

The structured questionnaire used in the survey included questions on socio-demographic factors, access to and utilization of health services, access to education, housing, sanitation, food security, savings and access to credits, and new information technologies. The list of questions used to collect data on healthcare use is presented in the annex (Appendix 1). The questionnaire was pretested before the data collection through a pilot test to assess the data collection and processing mechanisms (INSD, [18]).

### Data collection

Ninety-one (91) qualified data collectors were recruited in two phases. During the first phase, the shortlisted data collectors were trained during a workshop at the end of which, the most suitable candidates were selected and trained on the survey concept and rationale, questionnaire, data collection process, data quality assessment and ethical compliance. Data collectors were divided into three-person teams with an average of 30 clusters of 12 households per team led by a supervisor. Data were collected at the household level (respondents’ home) over 1 year (January to December 2014) during which data collectors visited the sampled households. For the respondents under the age of 15, their legal representative was interviewed.

Data collected were related to household consumption, which varies depending on the periods of the year (dry season, growing and harvest seasons). To capture these seasonal variations, data were collected every 3 months (four phases). Households that participated in at least three phases, were included in the dataset. Data entry was processed with the Census and Survey Processing System (CSPro). The dataset that we used, was anonymized.

### Ethical approval

The study used secondary data collected in a national household survey, which did not require ethical approval. The survey was conducted by a governmental institution, namely the National Institute of Statistics and Demography. As confirmed by the managers from the survey department of the institute, ethical approval was not required to carry out the survey.

### Data analysis

For our study, we used the data on households’ socio-demographic characteristics, utilization of health services and satisfaction with health services used. The following variables were included in the analysis (Appendix 2):

- *Dependent variables*
  - Service utilization in the preceding 15 days (used services or not)
  - Satisfaction with health services used in the preceding 15 days (satisfied or not)
  - Expenditures on health services (sum of all fees paid during the last episode of healthcare seeking). These included fees for consultation, medical checks, drugs, hospital admission, other expenditures and informal payments.
- *Independent variables*:
  - Age (categorized into six groups: 0–5, 6–14, 15–24, 25–39, 40–59 and ≥ 60 years)
  - Gender (male, female)
  - Residence (urban, rural)
  - Education (no education**,** primary, secondary, university)
  - Employment status (employed, self-employed and unemployed)
  - Distance to the health facility (< 1 km, 1–4 km, 5–9 km, ≥ 10 km).
  - Type of health facility (teaching hospitals, regional hospitals, district hospitals, medical centers, primary healthcare centers, private clinics, non-profit organizations, pharmacy and other health facilities). For the analysis, teaching hospitals and regional hospitals were categorized as referral hospitals. The medical centers were included in the category of district hospitals, the non-profit organizations and pharmacies were included in the category of other health facilities.
  - Income level: Given that data on income were not disaggregated to the individual level, the regional poverty index was used as a proxy to determine the individual income that was divided into three categories: high-income region, low-income region, very low-income region. The poverty index of each region was applied to all household members in the respective region.
  - Region: Administrative breakdown, which corresponds to the 13 regions of the country.

We carried out a regression analysis to determine the association between the dependent and independent variables listed above. Specifically, for the analysis of the first two dependent variables, the use of health services and satisfaction with services, we applied binary logistic regression and for the analysis of expenditures on health services, we applied linear regression.

In all three regressions, the set of independent variables included age, gender, residence, education, employment status, regional income level, and region. Distance to health facilities, and the type of health facilities were only included in case of satisfaction with services and expenditures on health services. In the case of health service use, we included respondents who were ill in the preceding 15 days, and in the case of satisfaction with services and expenditures on health services, we only included respondents who had used health services in the preceding 15 days. Data analyses were performed with the Statistical Package for the Social Sciences (SPSS) version 25. Associations were accepted to be significant when the *p*-value was smaller than 0.05 (95% CI).

## Results

From the initial sample of 10,800 households, 10,411 households (about 96%) participated in at least three phases of the data collection and were included in the dataset used for the analysis. The household sample included 72,401 individuals (households’ members). From those, a total of 10,009 individuals (about 13.82%) reported to have been ill in the preceding 15 days and were included in our analysis.

Table 1 presents the general socio-demographic characteristics of respondents (household members) included in the analyses. The under 5 years was the largest age group (31.20%) and older than 60 years was the smallest age group. More than half of the respondents (53.13%) were male. Most respondents (59.30%) were living in an urban area. With regard to education and employment status, the vast majority of respondents (78.12%) had no education and more than half (65.56%) were self-employed. On the income side, the classification showed that 79% of individuals were living in a very low-income region (high regional poverty index).

**Table 1:** Socio-demographic characteristics of respondents who were ill in the preceding 15 days (*N* = 10,009), Burkina Faso, 2014

Variables	Modalities	n (%)
Age group ^a^	0–5 years	2918 (31.20%)
	6–14 years	1916 (20.48%)
	15–24 years	1093 (11.68%)
	25–39 years	1449 (15.49%)
	40–59 years	1215 (12.99%)
	60 years +	763 (8.16%)
Gender	Male	5318 (53.13%)
	Female	4691 (46.87%)
Residence	Urban	5935 (59.30%)
	Rural	4074 (40.70%)
Education ^b^	No education	7240 (78.12%)
	Primary	1127 (12.16%)
	Secondary	751 (8.10%)
	University	150 (1.62%)
Employment status	Employed	2052 (20.50%)
	Self employed	6562 (65.56%)
	Unemployed	1395 (13.94%)
Regional income level based on the regional poverty index	High-income (well off regions)	2102 (21.00%)
	Low-income (poor regions)	4932 (49.28%)
	Very low-income (very poor regions)	2975 (29.72%)

^a^ missing data for 655 respondents; ^b^ missing data for 741 respondents

Table 2 presents the morbidity patterns among sick individuals by age group and gender. As reported by the respondents, the two most frequent diseases across all age and gender groups were malaria/fever (68.02 and 67.69% respectively) and diarrheas/stomach pains (9.23 and 9.44% respectively).

**Table 2:** Morbidity patterns among respondents who were ill in the preceding 15 days (*N* = 10,009)**,** Burkina Faso, 2014

	AGE GROUP n (%)														GENDER n (%)					
	0–5 years		6–14 years		15–24 years		25–39 years		40–59 years		60 years +		Total ^**a**^		Male		Female		Total ^**a**^	
Fever/Malaria	2370	(81.42%)	1477	(77.49%)	728	(66.91%)	881	(60.84%)	621	(51.11%)	270	(35.39%)	**6347**	**(68.02%)**	3317	(69.86%)	3440	(65.72%)	**6757**	**(67.69%)**
Diarrheas/Stomach pains	218	(7.49%)	146	(7.66%)	126	(11.58%)	194	(13.40%)	114	(9.38%)	63	(8.26%)	**861**	**(9.23%)**	402	(8.47%)	540	(10.32%)	**942**	**(9.44%)**
Back pains /Muscular or joint pains	16	(0.55%)	36	(1.89%)	45	(4.14%)	93	(6.42%)	186	(15.31%)	216	(28.31%)	**592**	**(6.34%)**	251	(5.29%)	371	(7.09%)	**622**	**(6.23%)**
Dentals pains/Teething problems	9	(0.31%)	15	(0.79%)	18	(1.65%)	36	(2.49%)	30	(2.47%)	12	(1.57%)	**120**	**(1.29%)**	46	(0.97%)	78	(1.49%)	**124**	**(1.24%)**
Skins disorders	47	(1.61%)	21	(1.10%)	7	(0.64%)	18	(1.24%)	12	(0.99%)	8	(1.05%)	**113**	**(1.21%)**	50	(1.05%)	77	(1.47%)	**127**	**(1.27%)**
Eye diseases	13	(0.45%)	14	(0.73%)	5	(0.46%)	7	(0.48%)	23	(1.89%)	28	(3.67%)	**90**	**(0.96%)**	46	(0.97%)	57	(1.09%)	**103**	**(1.03%)**
Blood pressure issues	2	(0.07%)	0	(0.00%)	5	(0.46%)	9	(0.62%)	39	(3.21%)	57	(7.47%)	**112**	**(1.20%)**	54	(1.14%)	61	(1.17%)	**115**	**(1.15%)**
Typhoid fever	5	(0.17%)	2	(0.10%)	0	(0.00%)	3	(0.21%)	1	(0.08%)	0	(0.00%)	**11**	**(0.12%)**	2	(0.04%)	10	(0.19%)	**12**	**(0.12%)**
Ear. Nose and throat diseases	79	(2.71%)	50	(2.62%)	26	(2.39%)	26	(1.80%)	19	(1.56%)	12	(1.57%)	**212**	**(2.27%)**	117	(2.46%)	120	(2.29%)	**237**	**(2.37%)**
Diabetes	10	(0.34%)	4	(0.21%)	4	(0.37%)	3	(0.21%)	6	(0.49%)	6	(0.79%)	**33**	**(0.35%)**	18	(0.38%)	17	(0.32%)	**35**	**(0.35%)**
Meningitis	3	(0.10%)	2	(0.10%)	1	(0.09%)	0	(0.00%)	0	(0.00%)	0	(0.00%)	**6**	**(0.06%)**	2	(0.04%)	5	(0.10%)	**7**	**(0.07%)**
Accident and injuries	34	(1.17%)	67	(3.52%)	36	(3.31%)	50	(3.45%)	35	(2.88%)	20	(2.62%)	**242**	**(2.59%)**	161	(3.39%)	99	(1.89%)	**260**	**(2.60%)**
Stomach ulcer	5	(0.17%)	0	(0.00%)	4	(0.37%)	22	(1.52%)	10	(0.82%)	2	(0.26%)	**43**	**(0.46%)**	15	(0.32%)	29	(0.55%)	**44**	**(0.44%)**
Others disease	100	(3.44%)	72	(3.78%)	83	(7.63%)	106	(7.32%)	119	(9.79%)	69	(9.04%)	**549**	**(5.88%)**	267	(5.62%)	330	(6.30%)	**597**	**(5.98%)**
**Total** ^**a**^	**2911**	**(100%)**	**1906**	**(100%)**	**1088**	**(100%)**	**1448**	**(100%)**	**1215**	**(100%)**	**763**	**(100%)**	**9331**	**(100%)**	**4748**	**(100%)**	**5234**	**(100%)**	**9982**	**(100%)**

^a^ Totals vary because of missing data, which are not reported in the table

Table 3 presents descriptive statistics on healthcare seeking behavior among the respondents who were ill in the 15 days preceding the survey. Data indicate that more than half of the respondents (59.30%) have sought care in health facilities. The data also show that 42.70% of the respondents traveled 1 to 4 km to obtain healthcare and almost three-quarter of the respondents lived within 5 km distance from the health facility. The most visited health facilities (81.39%) were the primary healthcare centers. About 89.55% of the healthcare users were satisfied with the care used. With regard to health expenditures, the data show that the fees incurred by the respondents during the last episode of healthcare seeking, ranged from $.004–$6000 and the average amount paid was estimated to be $28.

**Table 3:** Healthcare use among respondents who were ill in the preceding 15 days (*N* = 10,009), Burkina Faso, 2014

Variables	Modalities	n (%)
Use of health services (*n* = 10,009)	1 = Yes	5935 (59.30%)
	0 = No	4074 (40.70%)
Distance to health facilities (*n* = 5876) ^a^	Less than 1 km	1899 (32.22%)
	1 to 4 km	2509 (42.70%)
	5 to 9 km	940 (16.00%)
	10 km and More	528 (8.99%)
Type of health facility used (*n* = 5786) ^b^	Referral Hospitals	220 (3.80%)
	District Hospitals	567 (9.80%)
	Primary Healthcare Center	4709 (81.39%)
	Private Clinics	220 (3.80%)
	Others Health Facilities	70 (1.21%)
Patient satisfaction (*n* = 5935)	1 = Satisfied	5315 (89.55%)
	0 = Not satisfied	620 (10.45%)
Health expenditures (*n* = 5781) ^c, d^	Min – Max	$.004–$6000
	Mean (SD)	$28 ($163)

^a^ missing data for 59 respondents; ^b^ missing data for 149 respondents; ^c^ missing data for 154 respondents; ^d^ 1$ = 500 XOF

Tables 4 and 5 present the results of the regression analysis to investigate the association between the socio-demographic characteristics on the one side, and the use of health services, satisfaction with health services and expenditures on health services, on the other side.

**Table 4:** Binary logistic regression: Use of health services and satisfaction with health services, Burkina Faso, 2014

	Use of health services in the past 15 days ***0 = No; 1 = Yes***				Satisfaction with health services used ***0 = Not satisfied; 1 = Satisfied***			
Variables	B	S.E.	Sig.	Exp (B)	B	S.E.	Sig.	Exp (B)
Age group 0–5	−1.061	.087	.000	.346	1106	,179	,000	3021
Age group 6–14	−.461	.090	.000	.630	1006	,196	,000	2734
Age group 15–24	−.546	.100	.000	.579	,736	,208	,000	2087
Age group 25–39	−.573	.095	.000	.564	,586	,193	,002	1796
Age group 40–59	−.444	.097	.000	.642	,241	,190	,206	1272
*Age group 60 & plus (reference)*								
Gender (1 male, 2 female)	.039	.044	.376	1.040	,107	,097	,267	1113
Residence (1 urban, 2 rural)	.065	.050	.198	1.067	-,125	,122	,306	,883
Education (1 no education, 2 primary, 3 secondary, 4 university)	−.119	.037	.001	.888	-,121	,073	,097	,886
Employed	.141	.093	.131	1.151	,178	,188	,344	1195
Self Employed	.115	.084	.169	1.122	,204	,169	,227	1226
*Unemployed (reference)*					,178	,188	,344	1195
Regional poverty index (1 high income region, 2 low income region, 3 very low income region)	−.873	.126	.000	.418	-,269	,274	,326	,764
District hospitals					,143	,121	,240	1153
Primary health care facilities					,142	,072	,048	1152
Private clinics					,125	,080	,119	1133
Others health services					,062	,091	,499	1064
*Reference hospitals (reference)*								
Distance to health facilities (in km)					,019	,056	,729	1020
Region of Hauts Bassins	−.448	.118	.000	.639	− 1269	,242	,000	,281
Region of Boucle Mouhoun	1.571	.113	.000	4.810	-,335	,232	,148	,716
Region of Sahel	2.181	.221	.000	8.858	,348	,461	,451	1416
Region of Est	1.426	.224	.000	4.160	,017	,441	,969	1017
Region of Sud Ouest	1.575	.229	.000	4.833	-,441	,440	,317	,643
Region of Centre Nord	2.049	.221	.000	7.764	,226	,455	,619	1254
Region of Centre Ouest	1.375	.124	.000	3.954	-,109	,270	,686	,897
Region of Plateau Central	.672	.129	.000	1.958	,318	,290	,272	1375
Region of Nord	.840	.120	.000	2.316	,125	,255	,624	1133
Region of Centre Est	.652	.126	.000	1.919	-,557	,232	,017	,573
Region of Cascades	.200	.116	.085	1.221	-,626	,274	,022	,535
*Region of Centre – capital city (reference)*								
Constant	.966	.243	.000	2.626	1886	,577	,001	6595
N	9268				5285			
Nagelkerke R Square	.088				.060

**Table 5:** Linear regression: Expenditures on health services (in XOF; 1$ = 500 XOF), Burkina Faso, 2014

		Unstandardized coefficients	Standard error	Standardized coefficients	Significance
1	Constant	65,880,592	10,902,686		.000
	Age group 0–5	−21,728,797	5328,626	−.122	.000
	Age group 6–14	−18,986,395	5628,844	−.088	.001
	Age group 15–24	−15,867,354	6062,324	−.058	.009
	Age group 25–39	−12,242,446	5825,687	−.051	.036
	Age group 40–59	−13,486,427	5969,038	−.051	.024
	*Age group 60 & plus (reference)*				
	Gender (1 = male, 2 = female)	− 1997,472	2389,149	−.012	.403
	Residence (1 = urban, 2 = rural)	− 940,847	2976,286	−.005	.752
	Education (1 = no education, 2 = primary, 3 = secondary, 4 = university)	502,259	1912,551	.004	.793
	Employed	3891,259	5086,551	.019	.444
	Self Employed	1360,807	4601,008	.007	.767
	*Unemployed (Reference)*				
	District hospitals	−17,913,027	3626,737	−.125	.000
	Primary health care facilities	−13,016,295	2184,219	−.178	.000
	Private clinics	− 3498,486	2185,418	−.031	.109
	Others health services	− 8832,967	2514,848	−.056	.000
	*Referral hospitals (reference)*				
	Distance to health facilities (in km)	4292,265	1409,884	.046	.002
	Region of Boucle Mouhoun	−20,489,936	5893,710	−.069	.001
	Region of Sahel	−26,932,248	6592,869	−.075	.000
	Region of Est	−23,318,477	5960,513	−.078	.000
	Region of Sud Ouest	−17,411,574	6411,346	−.050	.007
	Region of Centre Nord	−23,419,934	6408,273	−.068	.000
	Region of Centre Ouest	−23,492,180	6467,619	−.065	.000
	Region of Plateau Central	−23,286,334	6239,037	−.071	.000
	Region of Nord	−18,696,484	5881,376	−.064	.001
	Region of Centre Est	−25,414,368	6067,694	−.082	.000
	Region of Cascades	−23,190,205	6414,380	−.065	.000
	Region of Centre Sud	−23,973,220	5880,809	−.082	.000
	Region of Hauts Bassins (Economical city)	−15,027,095	5913,452	−.050	.011
	*Region of Centre – capital city (reference)*				
	N	5781			
	R Square	.028			

Table 4 presents the association between the socio-demographic characteristics and use of health services, as well as the association between the socio-demographic characteristics and satisfaction with health services using a binary logistic regression. The results indicate a strong association between all age groups and the use of services (*p* < 0.0005), which decreases with an increase in age, showing thus that young individuals are the largest healthcare consumers. There is a strong negative association (*p* < 0.005) between education and the use of health services indicating that a higher level of education does not lead to greater use of health services. This finding was similar to that for income level where data showed that individuals with a high-income use health services less than those with low-income (*p* < 0.0005). The results show an association between almost all regions and the use of health services (*p* < 0.0005), excepted for the region of Cascades.

Among those who used health services, 89.55% were satisfied with the health services. This satisfaction was very strongly associated with the age group under 24 (*p* < 0.0005) and moderately with the age group of 25–39 (*p* < 0.005). Regarding the regions, an association was only found between the economic city of the country (region of Bobo-Dioulasso) and users’ satisfaction (*p* < 0.0005).

Table 5 presents the association between the respondents’ socio-demographic, economic characteristics and their expenditures on health services using a linear regression. The results indicate a strong association between the age group under 5 years and expenditures on health (*p* < 0.0005) and a moderate association for the age group of 6–14 (*p* < 0.005). These findings show that an increase in age was associated with a decrease in health expenditures, suggesting that household’s health expenditures were mostly on young children, which supported the hypothesis that children were the largest consumers of healthcare. Regarding the type of health facility, the results showed that health expenditures were strongly associated (*p* < 0.0005) with district hospitals, primary healthcare facilities and other health facilities (pharmacies, non-profit organizations). The distance traveled by respondents to health facilities was associated with health expenditures (*p* < 0.005) as well the vast majority of regions (*p* < 0.0005) except for the region of Hauts Bassins (economical city) and the region of Sud Ouest.

## Discussion

This study provides evidence on the health status and socio-demographic factors associated with health service use in Burkina Faso. The morbidity data from the household survey show that the main health problem in Burkina Faso is malaria. The high malaria morbidity is confirmed by the health management information system (HMIS) reports. In 2014, malaria represented 58.4% of all outpatients (HMIS^1^ report, 2014), which is comparable to our results. In 2018, the proportion of malaria was lower, 25.9% of all outpatients had malaria (Ministère de la Santé, [36]). The study’s results also show differences in health services use by socio-demographic characteristics of households surveyed.

Use of health services is higher among younger age groups than among the older age group. This finding is consistent with the official statistics, which indicate that young people were the largest healthcare consumers in 2014. In that year, children under 5 years made up 39% of outpatients in health facilities (Ministère de la Santé, [19]) and this proportion was estimated to 45% in 2018 (Ministère de la Santé, [36]).

Healthcare use is higher among the poorest. A study conducted in Zimbabwe (Zeng et al., [35]) found that the poorest households relied on primary healthcare facilities and the richest households had greater hospital utilization. In 2014 and 2018, the primary healthcare facilities represented 80% (Ministère de la Santé, [19]) and 88% (Ministère de la Santé, [36]) of public health facilities in Burkina Faso respectively. It could be argued that the greater use of health services by the population with low-income in our study is explained by the fact that the primary healthcare facilities are the most numerous (81.39%) and the most available source for healthcare seeking by the population, especially in rural areas. This is consistent with a study from Asia [22] that showed inequality in health centers utilization in favor of poorer households.

The results indicated that literate patients use health services less than those who are illiterate. These findings are similar to those from a household survey in rural Burkina Faso [20], which indicated that the proportion of people with no education who used health modern health services was 56.5% against 17.4% for those with primary education and 25.1% for those with secondary education. An interpretation of the negative education effect is that education makes households more efficient in maintaining health [12]. For instance, in 2015, nearly 72% of the population of Burkina Faso was living in rural areas (Ministère de l'Economie et des Finances, [17]) where the choice of healthcare facilities is limited to primary healthcare as an entry point into the health system. The relation between education and health could also suggest that improvements in education will raise the average income, make health services more affordable and equip people with the awareness needed to demand and obtain the health service they need [8].

Concerning users’ satisfaction, the main factor associated with being satisfied was the services provided during the visit to the health facility. Those who were not satisfied complained about the long waiting time, the low responsiveness of health personnel and the high cost of care. This is consistent with another study in Burkina Faso [26] on women’s satisfaction with delivery care, showing that 90% of service users were satisfied with the provider-patient interaction, the nursing care services and the environment. The satisfaction rates that we find are higher than those reported by another study on the quality of care of modern health services in Burkina Faso, which showed that 82.7% of users were satisfied with the way the medical doctors examined them (Baltussen & Ye, [3]). The dissatisfaction was related to the time spent to see a medical doctor. Similar to our findings, in Nigeria, it has been observed that a major group of respondents were satisfied with food and courtesy (53%), and with respect (61.5%) (Ogbeyi et al., [23]). Significant association was found with the age group of 35 to 44.

Four points have emerged from the households’ expenditures on health. The first point is the high health expenditures by the age group under 15. This could be explained by the fact that over half of sick people that have sought care in health facilities, was from this age group. A household survey in Sri Lanka [15] showed that the number of pre-school children is a stronger predictor of out-of-pocket payments than the number of elderly echoing Brown et al., [8] who suggests that pre-school children might need more preventive healthcare and often experience early-age illnesses.

The second point is the higher costs associated with the primary healthcare facilities and district hospitals. The higher costs observed in the primary healthcare facilities in our study are unexpected. The results of a study on out-of-pocket costs for facility-based maternity care in Burkina Faso, suggest that costs at health centers and dispensaries are less than those paid at hospitals [25]. The result observed in the study could be explained by the fact that the primary healthcare facilities were the first source of healthcare seeking (81.39%) with a proportional increase in health expenditures. The high costs associated with hospitals are consistent with the findings of several studies in Burkina Faso [16, 25], in Kenya and Tanzania [25]. These costs are related to the treatment of complicated cases referred in hospitals.

Thirdly, the distance traveled to health facilities was associated with health expenditures. In a study on households’ costs to severe malaria treatment in rural Burkina Faso, it was found that the main driver of the cost difference was the transportation [7]. Findings from a study in rural Bangladesh on the use of facility-based maternity services, showed that for each additional kilometer, the cost increased by $0–13 for antenatal care, $0–44 for delivery, and $0–11 for postnatal care [14]. According to the WHO, the transport costs can be even more prohibitive than the charges imposed for the services [34]. Beyond the cost, several studies have shown that distance is one of the key barriers that hamper access to health services in Burkina Faso and Sub Saharan countries [1, 4, 10, 21, 24] to the point that people cannot use services if they are not available close by, even if they are free of charge [34]. This requires particular attention, given that nearly 25% of the service users in our studies have traveled more than 5 km.

Finally, households’ expenditures were high in most of the regions and the capital city. This could be due to the urbanization of the region associated with the availability of diverse healthcare seeking sources, especially the private healthcare facilities compared to the other regions. A study conducted in Burkina Faso showed that patients in private facilities paid about 50% more for their drugs and 100% more for consultation fees than those in public facilities [5]. A national survey on out-of-pocket payments for maternal healthcare indicated that the amounts paid by women were higher in urban areas [16].

The study findings showed poor access and inequality in health services utilization in Burkina Faso where the UHC policy was accepted throughout the government and the health system. For the country to achieve UHC, policy makers, partners and other stakeholders should explore actions that guarantee that all people obtain the health services they need. UHC cannot be attained unless both health services and financial risk protection systems are accessible, affordable and acceptable [8]. To achieve both goals of health decentralization and the reforms are undertaken, the government needs to focus on policy interventions that make health services physically accessible, financially affordable and acceptable to the population.

### Study limitations

We need to acknowledge the limitations of our study. We used secondary data from the national households’ survey to carry out a regression analysis, which presents some limitations. First, we could not include all relevant variables but we had to limit our analysis to variables available in the dataset. Second, our study could only study associations between the households’ socio-demographic and economic characteristics, and their use, satisfaction and expenditures on health services. It was not possible to identify causal factors of access to healthcare in Burkina Faso. Third, the dataset included information on episodes of illness which were self-reported by the interviewees. We were unable to determine if those were clinically confirmed cases. Finally, the dataset did not contain sufficient data on the period of the year when the interview was conducted. Thus, we did not have data for the analysis of seasonal variability in morbidity and healthcare seeking behavior.

## Conclusion

Our study shows that primary healthcare facilities are the main source of care for households in Burkina Faso, particularly for young people and those with low-income. Despite a high level of service users’ satisfaction, the study has identified some reasons of dissatisfaction that can considerably discourage communities to use health facilities. Another key finding highlighted the high cost of healthcare driven by the youngers’ health demand and cost of transportation. Overall, the results of this study suggest that specific policies are needed to enhance geographical access to health services by reducing distance coverage, financial access through effective financial protection and to improve the quality of healthcare towards UHC.

## Supplementary Information

**Additional file 1: Appendix 1**. English wording of the questions used in the study

**Additional file 2: Appendix 2**. Study variables

## Data Availability

The datasets used and/or analyzed during the current study are available from the corresponding author on reasonable request.

## Footnotes

1 Health Management and Information System
