# Supplementary material for: Factors associated with access to healthcare in Burkina Faso: evidence from a national household survey
Source: BMC Health Serv Res. 2021 Feb 15;21:148. doi: 10.1186/s12913-021-06145-5 (PMC7885251; doi:10.1186/s12913-021-06145-5)
Supplement: Supplementary file 1 — Additional file 1: Appendix 1. English wording of the questions used in the study [file 12913_2021_6145_MOESM1_ESM.docx]

***Appendix 1. English wording of the questions used in the study***

**Socio-demographic characteristics**

- Age
- Occupation
- Residence
- Gender
- Education
- Socio-professional status
- Income level (region poverty index)

**Interview questions**

1. Did [householder member’s Name] has a health problem within the preceding 15 days?

2. What was the health problem/issue?

3. Did [householder member’s Name] sought care in health facilities or with a traditional healer?

4. For what reasons [householder member’s Name] has not sought care?

5. Where [householder member’s Name] has sought care [type of health facilities]?

6. Who [profile of health personnel] has seen [householder member’s Name] during health visit?

7. Did [householder member’s Name] was satisfied with the service provided at the health facilities?

8. What is the distance from [householder member’s Name] home to the health facility?

9. What was the total amount paid by [householder member’s Name] for consultation fees?

10. What was the total amount paid by [householder member’s Name] for laboratory check fees?

11. What was the total amount paid by [householder member’s Name] for drugs fees?

12. What was the total amount paid by [householder member’s Name] for hospital admission fees?

13. What was the total amount paid by [householder member’s Name] for others health expenses?

14. What was the total amount paid by [householder member’s Name] for informal payment?
