# Supplementary material for: Factors associated with access to healthcare in Burkina Faso: evidence from a national household survey
Source: BMC Health Serv Res. 2021 Feb 15;21:148. doi: 10.1186/s12913-021-06145-5 (PMC7885251; doi:10.1186/s12913-021-06145-5)
Supplement: Supplementary file 2 — Additional file 2: Appendix 2. Study variables [file 12913_2021_6145_MOESM2_ESM.docx]

***Appendix 2. Study variables***

- 1. **Dependent variables**

| **Variables** |  | **n (%)** | **Min., Max.** | **Mean (SD)** |
| --- | --- | --- | --- | --- |
| Use of health services | 1 = Yes | 5,935 (59.30%) | % |  |
|  | 0 = No | 4,074 (40.70%) | % |  |
| Patient satisfaction | 1 = Satisfied | 5,315 (89.55%) | % |  |
|  | 0 = Not satisfied | 620 (10.45%) | % |  |
| Health expenditures* | Sum (Total) |  | $.004-$6,000 | $28 ($163) |

***** Include: Total (sum) fees for consultation, medical checks, drugs, hospital admission, others expenditures

and informal payments

- 1. **Independent variables**

| **Variables** |  | **n (%)** | **Min., Max.** | **Mean (SD)** |
| --- | --- | --- | --- | --- |
| Age group | 1 = 0-5 yrs | 2 012 (36.50%) | % |  |
|  | 2 = 6-14 yrs | 1 060 (19.23%) | % |  |
|  | 3 = 15-24 yrs | 622 (11.28%) | % |  |
|  | 4 = 25-39 yrs | 825 (14.96%) | % |  |
|  | 5 = 40-59 yrs | 658 (11.94%) | % |  |
|  | 6 = 60 yrs & + | 336 (6.09%) | % |  |
| Gender | 1 = Male | 2 847 (47.99%) | % |  |
|  | 2 = Female | 3 086 (52.01%) | % |  |
| Residence | 1 = Urban | 2 134 (35.96%) | % |  |
|  | 2 = Rural | 3 801 (64.04%) | % |  |
| Distance from household to health facilities (services users) | 1 = Less than 1 km | 1 899 (33.06%) | % |  |
|  | 2 = 1 to 4 kms | 2 509 (42.70%) | % |  |
|  | 3 = 5 to 9 kms | 940 (16.00%) | % |  |
|  | 4 = 10 kms and More | 528 (8.99%) | % |  |
| Education | 1 = Not educated | 4 170 (76.37%) | % |  |
|  | 2 = Primary | 730 (16.37%) | % |  |
|  | 3 = Secondary | 473 (8.66%) | % |  |
|  | 4 = University | 87 (1.59%) | % |  |
| Socio professional status | 1 = Employed | 1 204 (20.29%) | % |  |
|  | 2 = Self employed | 3 885 (65.46%) | % |  |
|  | 3 = Unemployed | 846 (14.25%) | % |  |
| Type of health facilities (services users) | 1 = Referral Hospitals | 220 (3.80%) | % |  |
|  | 2 = District Hospitals | 567 (9.80%) | % |  |
|  | 3 = Primary Health Care Center | 4 709 (81.39%) | % |  |
|  | 4 = Private clinics | 220 (3.80%) | % |  |
|  | 5 = Others | 70 (1.21%) | % |  |
| Profile of care providers | 1 = Medical Doctor | 426 (7.22%) | % |  |
|  | 2 = Senior Nurse | 123 (2.08%) | % |  |
|  | 3 = Nurse | 5 133 (86.96%) | % |  |
|  | 4 = Pharmacist | 38 (0.64%) | % |  |
|  | 5 = Traditional Healers | 120 (2.03%) | % |  |
|  | 6 = Others health personnel | 63 (1.07%) | % |  |
| Individual income level (based on regions’ poverty index) | 1 = High income (Well off regions) | 1 332 (21.00%) | % |  |
|  | 2 = Low income (Poor regions) | 2 875 (48.44%) | % |  |
|  | 3 = Very low income (Very poor regions) | 1 728 (29.12%) | % |  |
